# Scalp Seeding Post Craniotomy and Radiosurgery for Solitary Brain Metastasis: A Case Report and Systematic Review

**DOI:** 10.7759/cureus.1083

**Published:** 2017-03-07

**Authors:** Waseem Sharieff, Liam Mulroy, Adrienne Weeks, Samina Mansoor, Rajbir Pahil, Muhammad U Islam

**Affiliations:** 1 Department of Radiation Oncology, Dalhousie University, Cape Breton Cancer Centre, Sydney NS; 2 Radiation Oncology, Dalhousie University, Cape Breton Cancer Centre, Sydney NS; 3 Neurosurgery, Capital Health District Authority, Dalhousie University; 4 Pathology, Capital Health District Authority, Dalhousie University; 5 Oncology, Dalhousie University, Cape Breton Cancer Centre, Sydney NS; 6 Radiology, Dalhousie University, Cape Breton Cancer Centre, Sydney NS

**Keywords:** brain metastases, scalp metastases, radiosurgery, craniotomy

## Abstract

**Background:**

Radiosurgery is being increasingly used post craniotomy for brain metastasis, instead of whole-brain radiation. We report a case of scalp metastasis following craniotomy and radiosurgery, along with a systematic review of the literature.

**Methods:**

Our patient was a 70-year-old male who presented with a scalp metastasis, two years after craniotomy and radiosurgery, for a solitary brain metastasis from esophageal carcinoma. Using Medline® (United States National Library of Medicine, Bethesda, MD), we performed a systematic review of the literature to identify cases of isolated scalp metastases following craniotomy for brain lesions.

**Results:**

The scalp metastasis was in close proximity to the craniotomy site. Workup did not show any other site of active disease. Biopsy confirmed it to be a metastasis from esophageal carcinoma. The literature review did not yield any case of isolated scalp metastasis following craniotomy and whole-brain radiotherapy or radiosurgery. However, it yielded six cases of isolated scalp metastases following craniotomy for primary brain tumors.

**Conclusion:**

Isolated scalp metastasis has not been reported following craniotomy and whole-brain radiotherapy for brain metastases. Our patient likely had surgical seeding during craniotomy. These surgically implanted cells could not be ablated because the radiosurgery treatment volume does not cover the surgical tract. Further research is needed to identify risk factors for surgical seeding.

## Introduction

For several decades, whole-brain radiation therapy (WBRT) remained the standard treatment for brain metastases. It was shown in a number of studies that surgery for solitary brain metastasis followed by WBRT resulted in improved overall survival compared to WBRT alone [1-4]. The introduction of radiosurgery allowed higher doses of radiation to the specified brain lesions with high precision [5]. When tested in limited brain metastases (one to three lesions), radiosurgery alone was associated with equivalent overall survival compared to radiosurgery plus WBRT [6-7]. This suggested that WBRT could be omitted in limited brain metastases that are treated with radiosurgery. Whether WBRT could be substituted with radiosurgery in post-surgical patients is not known. The National Cancer Institute of Canada (NCIC) is conducting a study (#CEC.3) specifically to address this question [8]. Patients are randomized to radiosurgery or WBRT following surgery for brain metastasis.

Although still experimental, sometimes patients are treated with radiosurgery off study out of their preference. We report a case of scalp metastasis following craniotomy and radiosurgery, along with a literature review.

Informed consent was obtained from the patient for this study.

## Materials and methods

### Case report

Our patient is a 70-year-old male. Initially, he presented in 2013 with progressive dysphagia. He was investigated with barium swallow and an esophago-gastroscopy. A tumor was identified at the gastroesophageal junction (GEJ); the biopsy showed moderately differentiated adenocarcinoma. He had further workup with computed tomography (CT)/positron emission tomography (PET) scan. Intense fluorine-18 (F-18) fluordeoxyglucose (FDG) uptake was noted at the GEJ and the proximal part of the lesser curvature of the stomach; a 7 mm lymph node in the gastro-hepatic ligament was mildly avid. The patient was staged as T2 N1 M0 and treated with neoadjuvant chemoradiation. Chemotherapy consisted of weekly carboplatin and paclitaxel. Radiation was delivered over five weeks at a dose of 4500 cGy/25 fractions to the tumor and regional lymph nodes. In July 2013, he underwent a total esophagectomy and proximal gastrectomy. The pathology revealed extensive fibrosis in the esophagus and stomach specimens consistent with post chemo-radiation changes. No viable or residual malignancy was identified. One out of nine perigastric lymph nodes was positive. The patient was treated with two cycles of adjuvant chemotherapy comprising of epirubicin, cisplatinum and 5-fluorouracil (ECF). He continued on surveillance with periodic gastroscopy.

**Figure 1:**
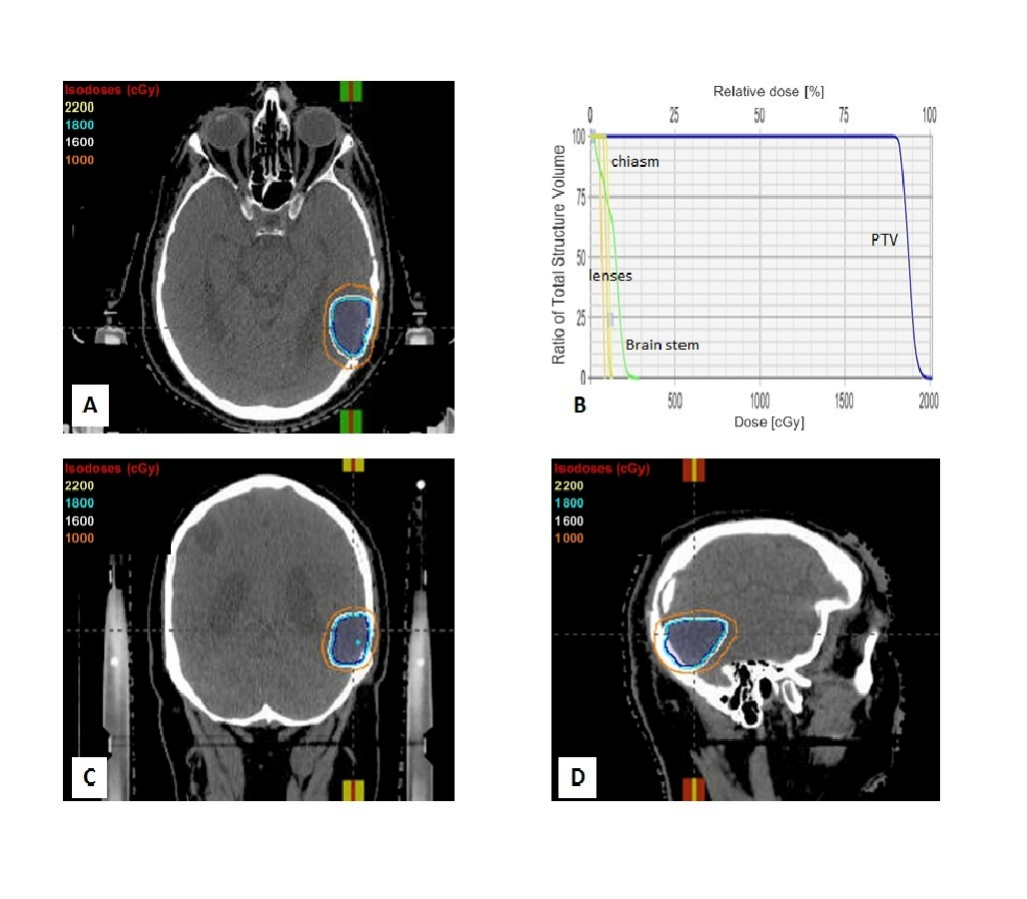
Radiosurgery Plan CT images showing planning target volume (PTV) and radiation isodose levels in axial (A), coronal (C) and sagittal (D) planes. Dose volume histogram showing adequate coverage of PTV while maximal sparing of the brain stem, chiasm, and lenses (B).

In December 2014, he presented with headaches. Magnetic resonance imaging (MRI) of the brain showed an enhancing cortically-based lesion with central necrosis in the left parieto-temporal region. It measured 4.1 x 3.7 cm and had surrounding vasogenic edema with mass effect on the lateral ventricle. He underwent a craniotomy and had an uneventful recovery. Pathology showed metastatic adenocarcinoma of esophageal origin. He received radiosurgery to the surgical cavity (1800 cGy single fraction) which was completed in February 2015 (Figure 1). Thereafter, he continued surveillance with three monthly MRI scans. After one year of surveillance, he was switched to six monthly MRI scans; he continued surveillance for systemic recurrence with CT chest, abdomen, and pelvis.

In April 2016, the MRI showed increased enhancement in the surgical cavity and a slight increase in edema in the left temporal lobe. Additionally, the MRI noted a new enhancing lesion to be arising from the deep portion of the scalp in the left parietal region. It measured 1.5 x 1.5 cm. The findings were concerning for disease recurrence.

Clinically the patient was doing well but a bony bulge was palpable over the left parietal skull. He was re-staged with a CT chest, abdomen, and pelvis and an MRI brain perfusion study was carried out. CT did not show any evidence of local recurrence or distant metastases. The MRI perfusion study did not show any significant blood flow in the surgical cavity but increased blood flow in the scalp lesion. This suggested radiation necrosis in the surgical cavity but active disease in the scalp. The lesion was biopsied and it confirmed the diagnosis of metastatic disease. Using a tangent pair of 6 MV photon beams, the left scalp lesion was treated with a 2 cm margin and 1 cm overlying bolus at a dose of 3250 cGy/5 fractions. For patient position verification, on-board kilovoltage images were used. These were overlaid over digitally reconstructed radiographs generated from the radiation plan. They were matched to the skull. For dose verification, in vivo dosimetry was employed by placing metal oxide semiconductor field effect transistors (MOSFETs) over the scalp lesion.

### Systematic review          

Using Medline® (United States National Library of Medicine, Bethesda, MD), we performed a search for publications on scalp metastases following surgery. Our disease-specific medical subject heading (MeSH) terms were ‘brain’, ‘neoplasm’, ‘metastases’ and ‘scalp’, and our treatment specific MeSH term was ‘craniotomy’. To keep the search exhaustive, we did not apply any filters for publication date and article type. Two of the authors independently performed the search and selected publications that met the eligibility criteria. We identified additional publications from the reference list of the selected publications. Eligibility criterion was any report of isolated scalp metastasis from surgical seeding. We defined surgical seeding as any scalp metastasis close to the surgical tract where the surgical cavity did not have any recurrent disease. We excluded cases of direct extension of recurrent tumor into the surgical tract, and recurrent tumor with multiple sites of metastases.

## Results

### Case report

**Figure 2:**
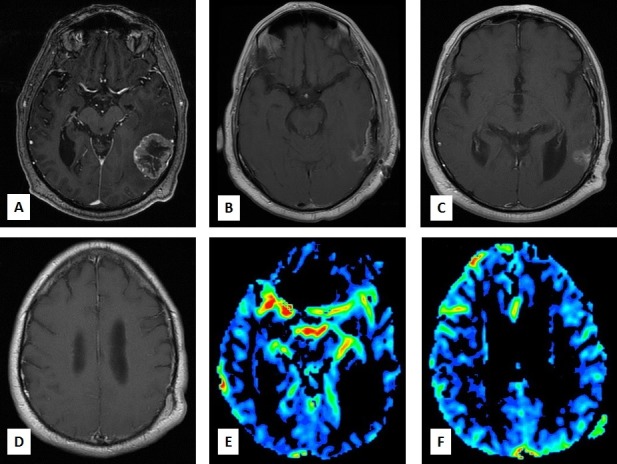
Scalp Metastasis Post Craniotomy and Radiosurgery A-D: Gadolinium enhanced T1 MRI sequences. A solitary metastatic lesion in the left parietal lobe is evident in A. B shows post-surgical changes following craniotomy. Enhancement in the surgical cavity is seen (C), and a new lesion in the left parietal bone is noted (D). E-F: MR perfusion images showing no blood flow in the surgical cavity (E), and increased blood flow in the region of scalp metastasis (F).

**Figure 3:**
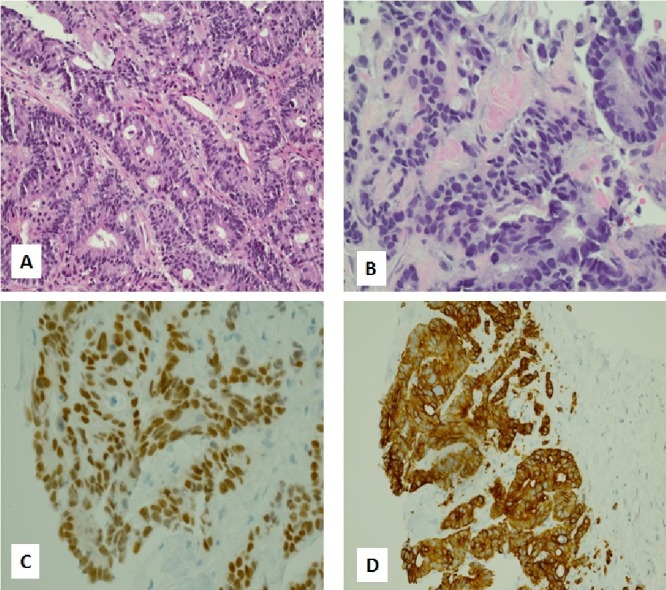
Scalp Metastasis From Esophageal Carcinoma A: Higher magnification of the primary tumor shows features of adenocarcinoma - glandular crowding, cribriforming, cytologic atypia, loss of polarity and increased mitotic activity. B: Higher magnification of scalp lesion shows similar features. C and D: Immunohistochemistry shows positive staining of cells to CDX2 and CK7, respectively.

**Figure 4:**
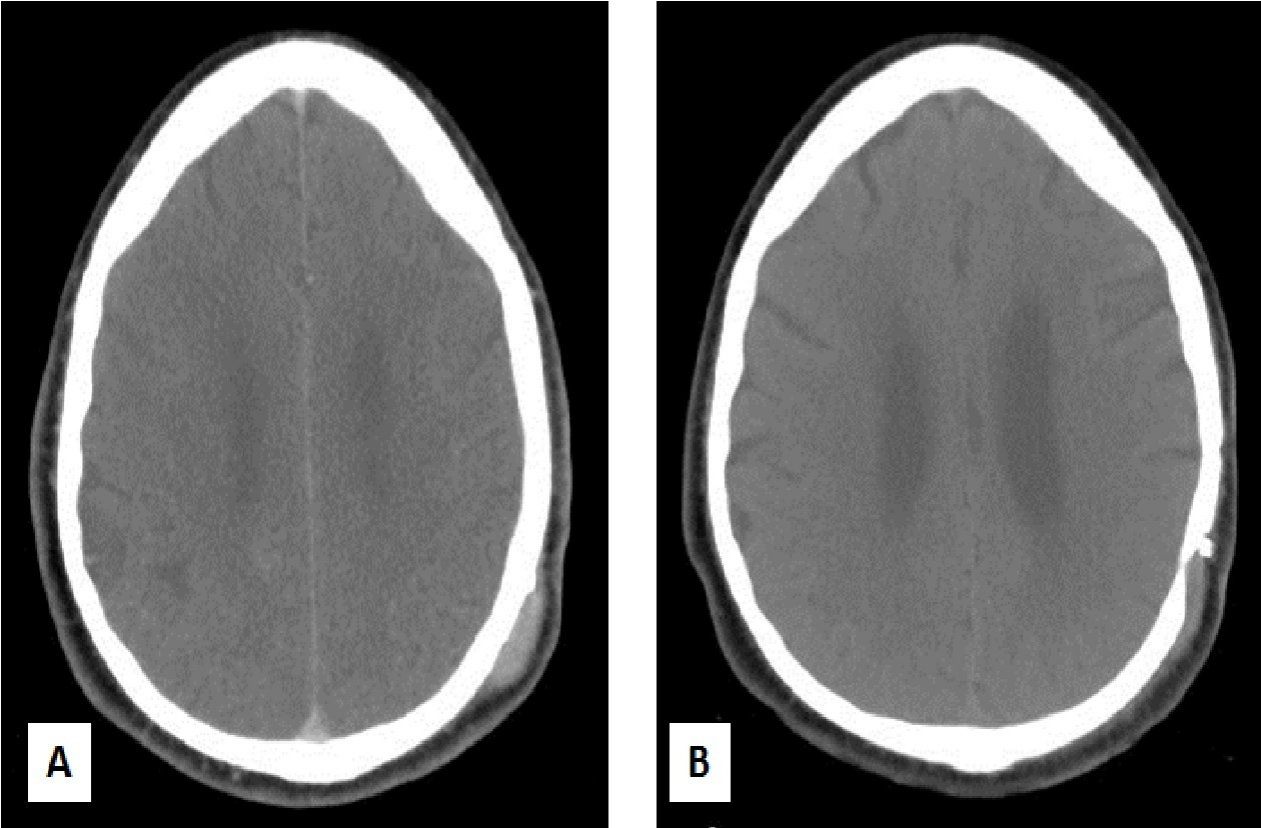
Scalp Metastasis A-B: Axial CT images showing scalp site of metastasis before (A) and after radiotherapy (B). Note the craniotomy defect.

The scalp metastasis was not present prior to craniotomy and appeared in close proximity to the craniotomy site (Figure 2). Biopsy showed fragmented portions of the epidermis and underlying dermis. Nests of tumor cells were seen in the dermis arranged in a glandular pattern. Immunohistochemical stains were positive for cytokeratin, CK7, and CDX2. This was consistent with an esophageal primary (Figure 3). The lesion responded to radiation therapy (Figure 4).

### Systematic review

The literature search identified 18 publications. A review of the reference list identified 15 additional publications. These reported a total of 27 cases of scalp metastases. Out of 27 cases, six met the eligibility criteria (Table 1). These comprised of primary brain tumors in which iatrogenic spread to scalp occurred during craniotomy [9-14]. The review did not yield any case of isolated scalp metastasis following craniotomy and whole-brain radiotherapy or radiosurgery. 

**Table 1:** Post Craniotomy Isolated Scalp Metastasis Reported in the Literature

Source	Primary site	Secondary site	No. of cases	No. of surgeries	Radiotherapy
Cappelaere, et al. 1972 [9]	Oligodendroglioma	Scalp	1	1	No
Chin, et al. 1980 [10]	Oligodendroglioma	Scalp	1	3	No
Ludemann, et al. 2002 [11]	Meningioma	Surgical scar	1	1	No
Ozer, et al. 2007 [12]	Meningioma	Pin site	1	2	Yes
Velnar, et al. 2008 [13]	Meningioma	Surgical scar	1	1	No
Tahir, e al. 2009 [14]	Meningioma	Surgical tract	1	1	No

## Discussion

We report a case of scalp metastasis following craniotomy and radiosurgery. To the best of our knowledge, this is the first report of scalp metastasis post radiosurgery. Radiosurgery was effective in achieving local control in the surgical cavity. However, it failed to prevent recurrence in the scalp. The likely mechanism of scalp metastasis was seeding during craniotomy.

Our literature review did not yield any case of isolated scalp metastasis following craniotomy for brain metastasis. This could be because a) in recent times, improvement in systemic therapy and aggressive treatment of oligometastases has considerably improved survival which was previously fairly low; b) brain metastasis was traditionally treated with whole-brain radiotherapy that might have sterilized the surgical tract; and c) surgical resection of brain metastasis recently became an established practice in select cases.

The differential diagnosis of scalp lesion includes hypertrophied scar, second primary malignancy, new metastasis, and recurrence from surgical seeding. Pathology proved it to be a metastasis of esophageal origin. Thus, it was either a new metastasis or recurrence from surgical seeding. Given that isolated metastasis to the scalp are extremely rare and re-staging workup did not reveal any other site of active disease, we believe that it was likely the result of surgical seeding during craniotomy.

Surgical seeding during craniotomy is uncommon. Very few cases have been reported which are mostly related to primary brain tumors. Avecillas-Chasin, et al. reported four cases of scalp metastases from recurrent meningiomas, along with the literature review [15]. They concluded that reoperations and torpid course of the surgical wound with cerebrospinal fluid fistula were among the few factors associated with scalp metastases.

The major limitation of our case report is that it is based on a single patient and thus the results are not generalizable to every case. Nevertheless, we included a systemic review which yielded six cases of isolated scalp metastasis likely from surgical seeding during craniotomy. This suggests that the risk of recurrence is not limited to the surgical cavity alone.

## Conclusions

In conclusion, the mechanism of scalp metastasis in our patient was likely surgical seeding during craniotomy. This occurred despite copious irrigation of the craniotomy site and the surgical tract. Since radiosurgery treatment volume did not include the surgical tract, it did not ablate surgically implanted cells. Further research is needed to identify risk factors for surgical seeding.
